# Shorter Incubation Period among COVID-19 Cases with the BA.1 Omicron Variant

**DOI:** 10.3390/ijerph19106330

**Published:** 2022-05-23

**Authors:** Hideo Tanaka, Tsuyoshi Ogata, Toshiyuki Shibata, Hitomi Nagai, Yuki Takahashi, Masaru Kinoshita, Keisuke Matsubayashi, Sanae Hattori, Chie Taniguchi

**Affiliations:** 1 Public Health Center of Neyagawa City, Neyagawa 572-0838, Japan; 2 Itako Public Health Center of Ibaraki Prefectural Government, Itako 311-2422, Japan; t.ogata@pref.ibaraki.lg.jp; 3 Public Health Center of Suita City, Suita 564-0072, Japan; shibata585@city.suita.osaka.jp (T.S.); matsubayashi542@city.suita.osaka.jp (K.M.); 4 Ibaraki Public Health Center of Osaka Prefectural Government, Ibaraki 567-8585, Japan; nagaihit@mbox.pref.osaka.lg.jp; 5 Fujiidera Public Health Center of Osaka Prefectural Government, Fujiidera 583-0024, Japan; takahashiyu@mbox.pref.osaka.lg.jp (Y.T.); kinoshitamas@mbox.pref.osaka.lg.jp (M.K.); 6 Ibaraki Prefetural Office, Mito 310-0852, Japan; sa.hattori@pref.ibaraki.lg.jp; 7 College of Nursing, Aichi Medical University, Nagakute 480-1195, Japan; amachi@kej.biglobe.ne.jp

**Keywords:** SARS-CoV-2, COVID-19, Omicron variant, incubation period, Alpha variant, Japanese Public Health Center, contact tracing

## Abstract

We aimed to elucidate the range of the incubation period in patients infected with the SARS-CoV-2 Omicron variant in comparison with the Alpha variant. Contact tracing data from three Japanese public health centers (total residents, 1.06 million) collected following the guidelines of the Infectious Diseases Control Law were reviewed for 1589 PCR-confirmed COVID-19 cases diagnosed in January 2022. We identified 77 eligible symptomatic patients for whom the date and setting of transmission were known, in the absence of any other probable routes of transmission. The observed incubation period was 3.03 ± 1.35 days (mean ± SDM). In the log-normal distribution, 5th, 50th and 95th percentile values were 1.3 days (95% CI: 1.0–1.6), 2.8 days (2.5–3.1) and 5.8 days (4.8–7.5), significantly shorter than among the 51 patients with the Alpha variant diagnosed in April and May in 2021 (4.94 days ± 2.19, 2.1 days (1.5–2.7), 4.5 days (4.0–5.1) and 9.6 days (7.4–13.0), *p* < 0.001). As this incubation period, mainly of sublineage BA.1, is even shorter than that in the Delta variant, it is thought to partially explain the variant replacement occurring in late 2021 to early 2022 in many countries.

## 1. Introduction

The SARS-CoV-2 variant B.1.1.529, referred to as the Omicron variant of concern (VOC), was first reported from South Africa [1] in late November 2021 and had rapidly spread to overtake the previously dominant Delta VOC in many countries by the end of 2021. Japan also experienced this rapid replacement of the Delta VOC, with Omicron reaching 92% of total newly-detected COVID-19 cases on 10–16 January 2022 [2].

There is some evidence of the transmissibility of Omicron leading to higher infectivity [3], lower efficacy of vaccination on preventing transmission [3] and higher reproduction number [4] than the Delta VOC. However, limited information on the incubation period of the virus in patients with Omicron is available. Therefore, we aimed to determine the range of incubation periods in patients with the SARS-CoV-2 Omicron VOC compared with the formerly predominant Alpha VOC in the Japanese population.

## 2. Materials and Methods

### 2.1. Subjects

The Japanese Public Health Center (PHC), a branch of local government, performs contact tracing of index cases of COVID-19 to identify the persons with whom the infected person was in close contact during their infectious period. The PHCs gather information on the time and setting, and the infected person’s close contacts. Individuals who had close contact with the index case are then required to be tested by polymerase chain reaction (PCR) at least once during a 7 to 14-day observation period from the time of diagnosis, according to the guidelines of the Infectious Diseases Control Law. This provides opportunities to identify subjects with symptomatic SARS-CoV-2 to enable the calculation of the incubation period.

Methods of selecting eligible subjects were described in the preceding study [5]. We collaborated with three PHCs (two in Osaka, one in Ibaraki, total residents: 1.06 million) to access data on the study subjects for the Omicron VOC. We collated close contacts who were diagnosed with symptomatic SARS-CoV-2 infection confirmed by PCR tests with a cycle threshold value of 40 or loop-mediated isothermal amplification test between 1 January and 28 January 2022, when the estimated probability of Omicron in incident SARS-CoV-2 infection was 95% in Japan [2]. In 1589 positive cases detected in the contact tracing, we extracted 172 individuals for whom we could estimate a single date of exposure and setting of the transmission without any other probable route of transmission (Group A).

For comparison with the Omicron VOC, we collaborated with four PHCs (three in Osaka, one in Ibaraki, total residents: 1.26 million) to examine the incubation period of the Alpha VOC. We collected close contacts who were diagnosed with symptomatic SARS-CoV-2 Alpha variant infection between 10 April and 10 May 2021, when the Alpha VOC in incident SARS-CoV-2 infection became dominant in Japan [6]. Of these, we extracted 51 individuals whose single date of exposure and setting of the transmission without any other probable route of transmission could be determined.

Informed consent was not required from the study subjects or their infectors because active epidemiological data analyses were performed in accordance with the Infectious Diseases Control Law. The Ibaraki Prefecture Epidemiological Research Joint Ethics Review Committee approved this study.

### 2.2. Data Items

We collected subjects’ demographic data, date of onset, date of the potential transmissible contact and place where the transmission occurred. The participating PHCs limited the eligible subjects who had no other apparent chance of close contact with SARS-CoV-2-positive individuals during the observation period. The incubation period was defined as the interval between the date of transmission and the date of onset of clinical symptoms/signs in the study subjects. History of receiving COVID-19 vaccine was obtained from PHC staff interviews of the study subjects. When the date of vaccination was 14 days prior to the date of the potential transmissible contact, we included this in the vaccine status.

### 2.3. Identification of the SARS-CoV-2 Omicron and Alpha Variants

The National Institute of Infectious Diseases, Tokyo (NIID) was monitoring the prevalence of the Omicron variant among SARS-CoV-2-positive individuals with L452R negativity in a nation-wide sampling of specimens. The prevalence was 95.2% (1311/1377) in those diagnosed between 28 December and 11 January, 99.3% (1291/1304) between January 12 and 17 and 99.98% (4282/4283) between January 18 and 24 in 2022 [2]. The NIID showed that 99.5% of the Omicron variants identified between the 52nd week in 2021 and the 4th week in 2022 in Japan were sublineage BA.1 [7]. Therefore, the L452R-negative SARS-CoV-2-positive individuals who were diagnosed during the above periods were assumed to have Omicron sublineage BA.1. The proportion of L452R mutation-negative cases among the nation-wide sample of SARS-CoV-2-positives diagnosed January 10–23 in 2022 was 95.4% (43,658/45,748) [2].

Identification of the Alpha variant was performed to identify N501Y mutation-positivity among SARS-CoV-2-positives. Because of the NIID, Tokyo had randomly selected N501Y mutation-positive samples to examine whole-genome sequences of the strain in Osaka and Ibaraki Prefecture in April and May 2021, and 100% of the samples tested there were the Alpha variant, we identified SARS-CoV-2 Alpha via the presence of this mutation. Identification of the L452R and N501Y mutation was accomplished by real-time one-step RT-PCR assay following the guidelines of the NIID, Tokyo.

### 2.4. Statistical Methods

The effects on the incubation period of SARS-CoV-2 vaccination (0 vs. twice or more) and the presence vs. absence of L452R were examined by Mann–Whitney U testing. Differences in age, vaccination and place of transmission were assessed by the Kruskal–Wallis test. We estimated median incubation time and important quantiles (5.0th, 25th, 75th and 95th percentiles) with a 95% confidence interval (CI) by fitting the parametric log-normal distribution in a Bayesian framework because this statistical technique for assessing incubation periods has been used for many other acute respiratory viral infections [8]. Statistical analyses were performed using R (version 4-1-1; The R Foundation for Statistical Computing, Vienna, Austria).

## 3. Results

All the study subjects were not inpatients nor immunocompromised persons. The mean ± SDM of the observed incubation period in Group A (*n* = 172) was 2.85 ± 1.20 days (Table 1). Age was not correlated with the length of the incubation period. Subjects who received COVID-19 vaccines twice or three times had significantly shorter mean incubation periods than those who were unvaccinated (2.65 days vs. 3.31 days, *p* < 0.001). The mean incubation periods were not significantly different according to the setting of the transmission. The distribution of the incubation periods among subjects who were determined to have the Omicron VOC was not significantly different from what was seen in L452R-unknown patients (Table 1). The mean incubation period of the Omicron VOC was significantly shorter than in 51 COVID-19 patients with the Alpha VOC (3.03 ± 1.33 vs. 4.94 ± 2.19 days, *p* < 0.001).

**Figure 1:**
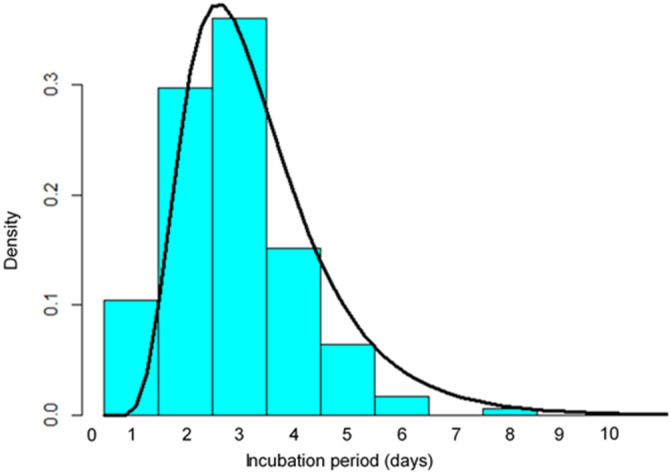
Probability density of the incubation period of SARS-CoV-2 using the log-normal distribution (solid line). Data are from 172 cases diagnosed in January 2022 when the Omicron VOC became predominant in Japan (dark bars).

Figure 1 shows the observed probability density of the incubation period in these 172 study subjects (Group A) by fitting the probability density of log-normal distribution. The highest density was observed in subjects who had a three-day incubation period. The estimated median incubation period in the log-normal distribution model (μ = 0.95, σ = 0.46) was 2.6 days (95% confidence interval (CI): 2.4 to 2.8) (Table 2). The estimated 5th and 95th percentiles were 1.2 days (95% CI 1.0 to 1.6) and 5.5 days (95% CI 4.7 to 6.5), respectively.

**Figure 2:**
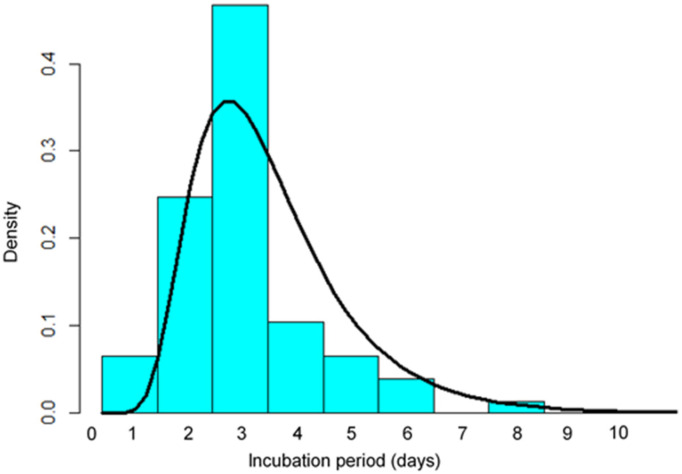
Probability density of the incubation period of 77 COVID-19 patients with the Omicron VOC using the log-normal distribution (solid line). Data are from 77 cases diagnosed in January 2022 in Japan (dark bars).

**Figure 3:**
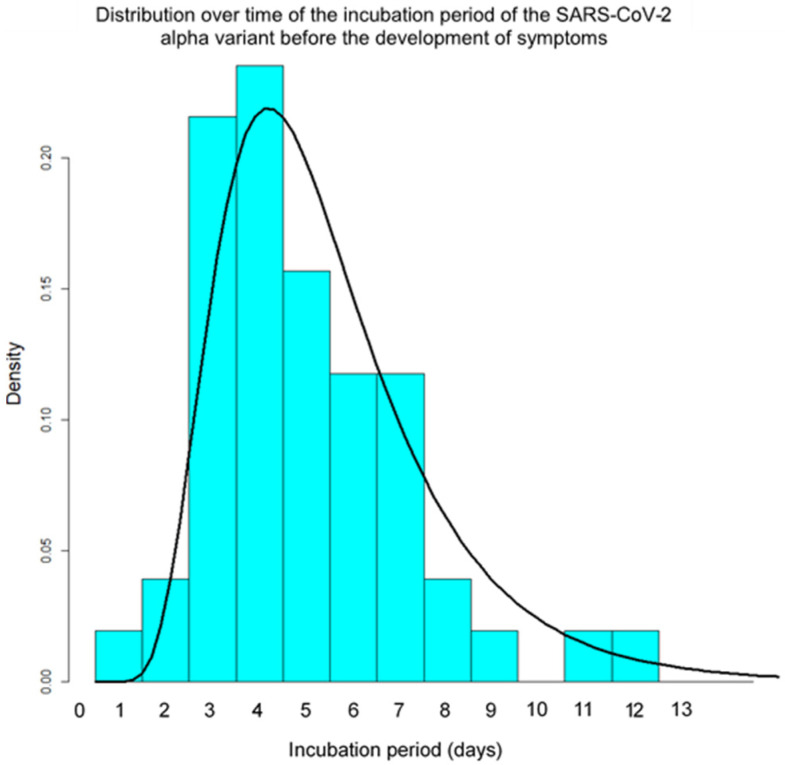
Probability density of the incubation period of the Alpha variant of SARS-CoV-2 using the log-normal distribution (solid line) in 51 cases diagnosed between 10 April and 10 May 2021 in Japan.

A similar probability density of the incubation period in 77 Omicron-positive individuals indicated a maximum density at day three (μ = 1.02, σ = 0.45, Figure 2). The estimated 5th, 25th, 50th, 75th and 95th percentiles were 1.3, 2.0, 2.8, 3.8 and 5.8 days (Table 2). This probability density was shifted towards being shorter than among the 51 cases with the SARS-CoV-2 Alpha variant (μ = 1.50, σ = 0.46, Figure 3). The estimated 5th, 25th, 50th, 75th and 95th percentiles of the incubation period among these 51 cases were 2.1, 3.5, 4.5, 6.1 and 9.6 days, respectively (Table 2).

## 4. Discussion

This study found that the estimated median incubation period in cases infected with the Omicron variant of COVID-19, mainly sublineage BA.1, was 2.8 days, with the vast majority falling between 1 and 6 days according to a log-normal distribution. The distribution was significantly shorter than that in patients with the Alpha variant, for which the estimated median period was 4.5 days.

Estimates in a meta-analysis using 34 publications of the incubation period of the ancestral virus were a mean of 6.3 and median of 5.4 days [9]. Our preceding study using contact tracing data estimated the median incubation period of the Delta variant was 3.7 days (95%CI = 3.3–3.7) [5]. These studies demonstrate that the incubation period of the SARS-CoV-2 Omicron variant is shorter than that of the preceding SARS-CoV-2 variants. Shorter serial intervals in SARS-CoV-2 cases with Omicron BA.1 variant compared with Delta variant were reported in the Netherlands [10]. As the length of incubation period is linked to the length of serial interval, the shorter incubation period is thought to partially explain the replacement of the Alpha with Delta variant in early to mid-2021 and the Delta with the Omicron variant in late 2021 to early 2022.

Mutations of the omicron variant confer improved stability and enhance attachment [11], which is thought to contribute to its higher transmissibility relative to the ancestral viruses [3]. This increased affinity could result in more rapid development of high viral loads, resulting in a shorter incubation period because the viral load in COVID-19 patients is likely to be inversely correlated with the incubation period [12].

Our findings indicate that COVID-19 cases who had received a SARS-CoV-2 vaccine more than once had shorter incubation periods than those who were unvaccinated. This may be because recipients of multiple vaccines retain immunological memory against SARS-CoV-2 antigens for longer periods, and thus induce inflammatory reactions more rapidly than those receiving a single vaccination or no vaccine. To verify this notion, further epidemiological studies considering some confounding factors are required.

Our study has some limitations. We selected patients with COVID-19 for whom the date and setting of infection were determined without any other probable route of transmission through contact tracing of their index cases. As this investigation was partially dependent on interviewing the SARS-CoV-2-positive individuals and their contacts, the possibility of recall bias cannot be excluded. The relatively small sample size also resulted in a wide range of estimates for the incubation period. We could not obtain information on the type of vaccine in the contact tracing data, which might influence the length of the incubation period among the vaccinated cases. Finally, the subjects with the Omicron VOC were recruited in January 2022, before Omicron sublineage BA.2 infection was increasing in Japan. As the effective reproduction number of Omicron BA.2 is larger than that of Omicron BA.1 [13], we need to study viral dynamics on the sublineage BA.2.

## 5. Conclusions

This study found that the estimated median incubation period in patients infected with the Omicron variant, mainly sublineage BA.1 of SARS-CoV-2, was around 2.8 days, with the vast majority falling between 1 and 6 days. This is significantly shorter than for the Alpha variant (median: 4.5 days).

## Figures and Tables

**Table 1:** Incubation period among Japanese COVID-19 patients diagnosed in January 2022.

		*n*	Mean	SD	*p*-Value
Total		172	2.85	1.20	
Age	0–9	12	3.00	1.00	0.368 ^(a)^
	10–19	23	2.91	0.78	
	20–39	91	2.90	1.14	
	40–59	13	3.23	2.12	
	60-	33	2.45	1.05	
Vaccination status	Unvaccinated	45	3.31	1.23	0.00416 ^(a)^
	Dose 1	3	2.33	0.943	
	Dose 2 or 3 times	118	2.65	1.25	
	Unknown	6	3.50	1.26	
Place	Restaurant/Karaoke/Bar/Party	110	2.94	1.24	0.0588 ^(a)^
	Home/Car	5	2.60	1.36	
	Sports activity	20	3.25	0.77	
	Nursing home	18	2.33	1.20	
	Nursery school	10	2.70	1.00	
	Other/Unknown	9	2.22	0.92	
variant	L452R(-)	77	3.03	1.33	0.118 ^(b)^
	Untested/Unknown	95	2.71	1.06	

(a) Kruskal–Wallis test, (b) Mann–Whitney U test, SD: standard deviation.

**Table 2:** Percentiles of the log-normal distribution of the incubation period based on the COVID-19 patients.

Percentile	Group A (*n* = 172)		Omicron VOC (*n* = 77)		Alpha VOC (*n* = 51)	
	Incubation Period (Days)	[95%CI]	Incubation Period (Days)	[95%CI]	Incubation Period (Days)	[95%CI]
5th	1.2	[1.0–1.6]	1.3	[1.0–1.6]	2.1	[1.5–2.7]
25th	1.9	[1.7–2.1]	2	[1.7–2.4]	3.5	[2.7–3.9]
50th	2.6	[2.4–2.8]	2.8	[2.5–3.1]	4.5	[4.0–5.1]
75th	3.5	[3.2–3.9]	3.8	[3.3–4.4]	6.1	[5.1–7.5]
95th	5.5	[4.7–6.5]	5.8	[4.8–7.5]	9.6	[7.4–13.0]

Group A: Japanese symptomatic COVID-19 patients who were diagnosed between 1 January and 28 January 2022. They included 77 cases with Omicron VOC. CI: confidence interval. VOC: variant of concern.

## Data Availability

Not applicable.
